# Comparative Omics Analysis of Historic and Recent Isolates of *Bordetella pertussis* and Effects of Genome Rearrangements on Evolution

**DOI:** 10.3201/eid2701.191541

**Published:** 2021-01

**Authors:** Ana Dienstbier, Fabian Amman, Denisa Petráčková, Daniel Štipl, Jan Čapek, Jana Zavadilová, Kateřina Fabiánová, Jakub Držmíšek, Dilip Kumar, Mark Wildung, Derek Pouchnik, Branislav Večerek

**Affiliations:** Institute of Microbiology of the Czech Academy of Sciences, Prague, Czech Republic (A. Dienstbier, D. Petráčková, D. Štipl, J. Čapek, J. Držmíšek, D. Kumar, B. Večerek);; Institute for Theoretical Chemistry of the University of Vienna, Vienna, Austria (F. Amman);; National Institute of Public Health, Prague (J. Zavadilová, K. Fabiánová);; Washington State University, Pullman, Washington, USA (M. Wildung, D. Pouchnik)

**Keywords:** Bordetella pertussis, bacteria, pertussis, whooping cough, omics analysis, insertion sequence element, genomic rearrangements, pathogen evolution, Czech Republic

## Abstract

Despite high vaccination coverage, pertussis is increasing in many industrialized countries, including the Czech Republic. To better understand *Bordetella pertussis* resurgence, we analyzed historic strains and recent clinical isolates by using a comparative omics approach. Whole-genome sequencing showed that historic and recent isolates of *B. pertussis* have substantial variation in genome organization and form separate phylogenetic clusters. Subsequent RNA sequence analysis and liquid chromatography with mass tandem spectrometry analyses showed that these variations translated into discretely separated transcriptomic and proteomic profiles. When compared with historic strains, recent isolates showed increased expression of flagellar genes and genes involved in lipopolysaccharide biosynthesis and decreased expression of polysaccharide capsule genes. Compared with reference strain Tohama I, all strains had increased expression and production of the type III secretion system apparatus. We detected the potential link between observed effects and insertion sequence element–induced changes in gene context only for a few genes.

*Bordetella pertussis* is a gram-negative, strictly human pathogen of the respiratory tract and the major causative agent of whooping cough. This highly contagious disease is especially severe in infants and remains a major cause of infant illness and death worldwide, predominantly in industrialized countries (*1*). Although pertussis is a vaccine-preventable disease, increased incidence is being observed in some countries that have highly vaccinated populations, including the Czech Republic (*2**–**4*). Although several factors are contributing to pertussis resurgence in these countries (*5**–**7*), the 2 prominent factors are incomplete and short-lived immunity induced by current acellular vaccines (*8**–**10*) and genetic variation, leading to escape from immunity by antigenic variation (*11**–**13*).

*B. pertussis* has an efficient mechanism of genome structure diversification because it contains >200 copies of insertion sequence 481 (IS481) in its genome (*14*). IS element–mediated homologous recombination results in excision or insertion of flanking genome regions and leads to genome reduction and decay (*14**–**16*), as well as genome rearrangements (*17**,**18*) and large duplications (*19*). Furthermore, a previous study indicated that gene order rearrangements associated with IS elements can alter gene expression profiles in *B. pertussis* (*20*). Recently, we have shown that, besides their effect on genome structure and stability, ISs can affect expression profiles of neighboring genes by IS element–specific promoters (*21*).

On the basis of these observations, we hypothesized that strains with different genomic organization should display altered global transcriptomic and, consequently, proteomic profiles, and thereby genome rearrangements might contribute to strain variation and adaptation. To validate this assumption, we have performed genomic, transcriptomic, and proteomic analyses of recent clinical isolates from the Czech Republic obtained during 2008–2015, previously characterized vaccine strains isolated during 1954–1965 (*22*) (hereafter referred to as historic strains), and the reference strain Tohama I.

## Materials and Methods

### Bacterial Strains and Growth Conditions

Recent isolates of *B. pertussis* from the Czech Republic were obtained from the National Institute of Public Health in Prague (Table 1). Historic strains from the Czech Republic (*22*) and reference strain Tohama I (*23*) have been described. All strains were cultivated on Bordet-Gengou agar plates supplemented with 15% sheep blood for 3–4 days at 37°C. For liquid cultures, bacteria were grown in Stainer-Scholte medium (*24*) supplemented with 0.1% cyclodextrin and 0.5% casamino acids (Difco, https://www.fishersci.com) at 37°C. To harvest samples for DNA, RNA, and protein isolation, *B. pertussis* cells were grown overnight in Stainer-Scholte medium to mid-exponential phase of growth (optical density ≈1.0). Three independent cultivations were performed to collect 3 biologic replicates of each of the strains for RNA and protein isolation.

**Table 1:** Characteristics of recent isolates of *Bordetella pertussis* and 9 infected patients, Czech Republic*

Year	Strain information			Patient information	
	Name	Genotype		Age, y/sex	Vaccination status
2008	Bp155	*ptxP3, fim2–1, fim3B, prn2*		<1/M	Not vaccinated
2008	Bp312	*ptxP3, fim2–1, fim3B, prn2*		45/F	wP
2012	Bp6260	*ptxP3, fim2–1, fim3A, prn2*		<1/F	Not vaccinated
2012	Bp6242	*ptxP3, fim2–1, fim3B, prn2*		67/F	Not vaccinated
2012	Bp6384	*ptxP3, fim2–1, fim3A, prn2*		69/M	Not vaccinated
2012	K10	*ptxP3, fim2–1, fim3B, prn3*		8/F	aP
2014	Bp82	*ptxP3, fim2–1, fim3A, prn2*		14/F	wP plus aP
2014	Bp46	*ptxP3, fim2–1, fim3A, prn2*		15/M	wP plus aP
2015	Bp318	*ptxP3, fim2–1, fim3A, prn2*		7/F	aP

*aP, acellular vaccine; wP, whole-cell vaccine.

### Genomic Analyses

For the genome organization analysis, genomic sequences were aligned by using the progressive Mauve algorithm (*25*) and clustered on the basis of their genome organization similarity by using the maximum-likelihood for the gene order pipeline (*26*). For single-nucleotide polymorphism (SNP) analysis, IS elements within the genomes were masked with Ns, and resulting sequences were aligned by using Mugsy software (*27*). SNPs were extracted by using custom scripts (https://genohub.com). Maximum-parsimony phylogenetic analysis was performed on sequences with masked IS elements by using the kSNP3 program with a k number of 23 (*28*). The unrooted phylogenetic tree was visualized by using iTOL (*29*).

### RNA Isolation, Sequencing, and Data Analysis

We provide information on RNA isolation, sequencing, and data analysis (Appendix 1). RNA sequencing data from sequencing runs were deposited in the European Nucleotide Archive under project accession no. PRJEB34096. We defined significance as a q value <0.05 (p value adjusted for multiple testing correction [Appendix 1]).

### Protein Sample Preparation and Proteomic Analysis 

We compiled information on protein sample preparation and label-free proteomic analysis, which used liquid chromatography with mass tandem spectrometry analyses (Appendix 1). Proteomics data were deposited in the ProteomeXchange Consortium by using the PRIDE partner repository with the dataset identifier PXD015184.

## Results

### Genome Organization and Content of Recent Isolates

**Figure 1:**
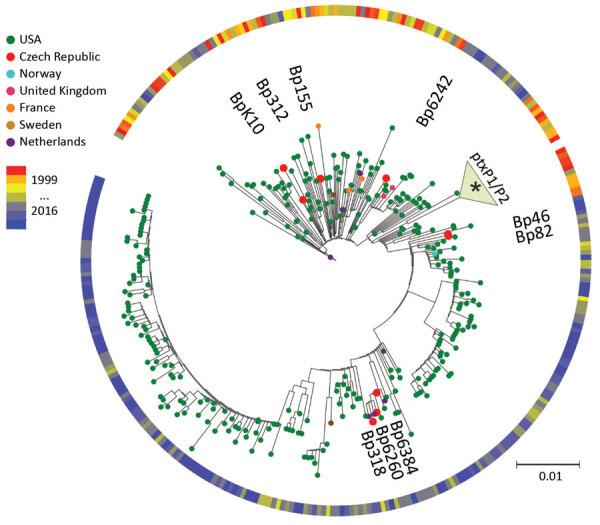
Maximum-parsimony, unrooted phylogenetic tree based on single-nucleotide polymorphism analysis of available genome sequences of *Bordetella pertussis*. Red dots indicate recent isolates from the Czech Republic. Year and country of isolation are color-coded. The 3 black dots indicate time span between 1999 and 2016. Asterisk (*) indicates association of historic strains from the Czech Republic with the *ptxP1*/*ptxP2* clade. Scale bar indicates nucleotide substitutions per site.

**Figure 2:**
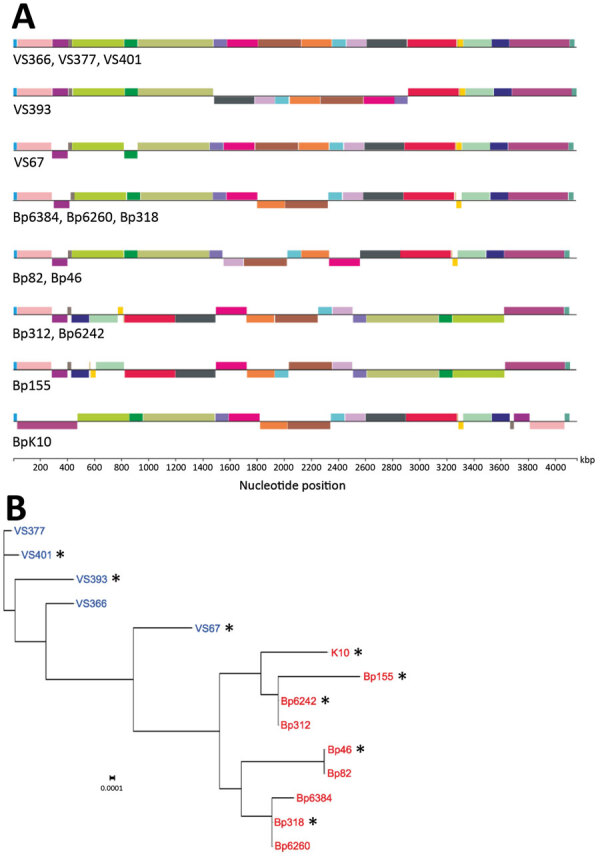
Genomic analyses of *Bordetella pertussis* isolates from the Czech Republic. A) Genome alignment of historic and recent isolates showing large-scale genome rearrangements. Homologous gene blocks are denoted by the same color. B) Maximum-likelihood phylogenetic tree based on genomic organization of historic (blue) and recent (red) isolates. Asterisk (*) indicates strains selected for transcriptomic and proteomic analyses. Scale bar indicates nucleotide substitutions per site. kbp, kilobasepairs.

We determined complete de novo genome assemblies of 9 recent isolates of *B. pertussis* strains collected in the Czech Republic during 2008–2015 from patients representing different age groups and vaccination status (Table 1). Genotyping of recent strains showed that they belonged to *ptxP3* lineage. SNP-based phylogenetic analysis of these strains and >350 complete *B. pertussis* genome sequences currently deposited in GenBank (Appendix 2 Table 1) showed that recent *B. pertussis* isolates cluster with *ptxP3* isolates from other countries, demonstrating worldwide spread and lack of geographic signature (Figure 1). The genome alignment of recent isolates and previously characterized historic strains belonging to the *ptxP1* lineage (*22*) showed that all genomes contain large-scale structural rearrangements (Figure 2, panel A). According to their genome organization, sequenced strains could be classified into 8 groups. None of the historic strains clustered with any of the recent isolates. The separation of these 2 groups was verified by using a maximum-likelihood phylogenetic tree, which was constructed on the basis of the genome organization of all sequenced strains from the Czech Republic (Figure 2, panel B).

To check whether there are also sequence signatures differentiating these 2 groups of strains, we performed SNP analysis, which yielded 35 SNPs (15 synonymous, 13 nonsynonymous, and 7 intergenic) (Appendix 2 Table 2) and distinguished historic and recent isolates. Variants found in historic strains were also present in Tohama I. One of the new SNPs specific for recent isolates was identified in the promoter region of the *bteA* gene, which encodes the type III secretion system (T3SS) effector. Approximately one third of the SNPs have been reported to be specific for the *ptxP3* lineage isolates from other countries (*30*). When compared with those of historic strains, the genome size of recent isolates was substantially reduced, thereby confirming ongoing gene loss within the global population of *B. pertussis* (Appendix 2 Table 3). Analysis of genome alignments showed 2 regions of difference (RD) between the 2 groups of strains from the Czech Republic, which corresponded to previously identified regions RD3 and RD10 (*20*). Although RD3 (28.7 kb, spans genes *BP0910A–BP0937*) is absent in all recent isolates, the RD10 (25.1 kb, spans genes *BP1948–BP1968*) is absent in all recent isolates and historic strain V67.

### Transcriptomic Profiles and Genomic Structure Alterations

**Figure 3:**
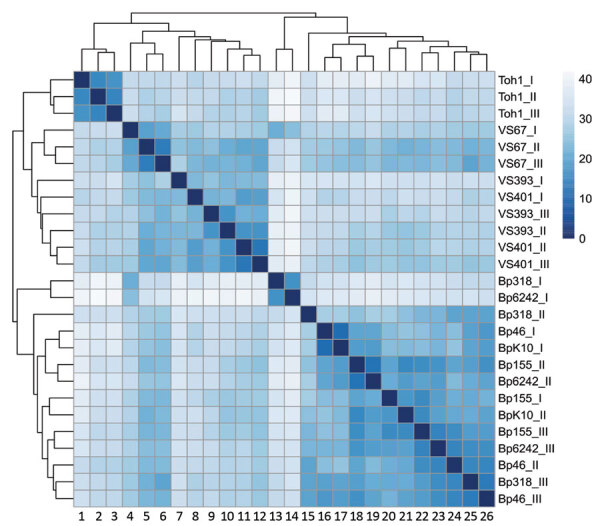
Heat map of sample-to-sample distances showing the hierarchical clustering of historic and recent isolates of *Bordetella pertussis* from the Czech Republic and the Tohama I strain. Colored scale bar and numbers on the right indicate Euclidian distances between samples calculated by using rlog-normalized RNA sequence data. 1, Toh1_1; 2, Toh1_II; 3, Toh1_III; 4, VS67_1; 5, VS67_II; 6, VS67_III; 7, VS93_I; 8,VS401_I; 9,VS393_III; 10, VS393_II; 11, VS401_II; 12,VS401_III; 13, Bp318_I; 14, Bp6242_I; 15, Bp318_II; 16, Bp46_I; 17, BpK10_I; 18, Bp155_II; 19, Bp6242_II; 20, Bp155_I; 21, BpK10_II; 22, Bp155_III; 23, Bp6242_III; 24, Bp46_II; 25, Bp318_III; 26, Bp46_III.

Total RNA was isolated from biologic triplicates of *B. pertussis* Tohama I strain; historic strains VS393, VS67, and VS401; and recent isolates Bp318, Bp155, Bp46, Bp6242, and BpK10 and analyzed by using RNA sequencing. These strains were selected on the basis of genome organization and phylogenetic distances to encompass the highest variability among the studied strains (Figure 2, panel B). Hierarchical clustering of RNA sequence data showed that samples from both groups of strains from the Czech Republic clustered separately from each other and from Tohama I (Figure 3). Consistent with phylogenetic analysis (Figure 2, panel B), we found that samples of strain VS67 formed a separate cluster. These analyses suggested that among historic strains, the VS67 strain displays closest distance to recent strains, which is consistent with our previous observation that the VS67 strain clusters together with a recent U.S. isolate (*22*).

Differential expression (DE) analysis identified 78, 124, and 115 significantly (q<0.05 for all comparisons) modulated *B. pertussis* genes (–1>log_2_ FC>1) between recent isolates and historic strains, recent isolates and Tohama I, and historic strains and Tohama I, respectively (Appendix 2 Table 4). Among the DE genes, 30 were up-regulated in recent isolates compared with historic strains, including those encoding the flagella apparatus (*flgB*-*J*), LuxR (*BP1969*), and ArsR (*BP2946*) families of transcriptional regulators, phosphoglucomutase (*pgm*), phosphoglucose isomerase (*pgi*), and nicotinate-nucleotide diphosphorylase (*nadC*). Conversely, among the 48 DE genes down-regulated in recent isolates were genes encoding the polysaccharide capsule proteins (*kpsEMT*), several ABC transporters, and central metabolism enzymes, including those involved in tryptophan synthesis (*trpDEG*). Expression of several virulence factors, including pertactin, tracheal colonization factor, filamentous hemagglutinin, and pertussis toxin subunit S3, was significantly up-regulated in recent isolates. However, the increase did not reach the 2-fold threshold. A recent isolate-specific SNP, which was identified in the promoter region of the *bteA* gene, did not result in a significant change of gene expression (Appendix 2 Table 4). Among the DE genes that showed increased expression in both groups of strains from the Czech Republic compared with Tohama I, we identified numerous genes within the T3SS *bcs*/*btr* locus, including *bsp22*, *bopN*, *bopB*, and *bopD* and several genes involved in sulfate metabolism (*cysADITW*).

**Figure 4:**
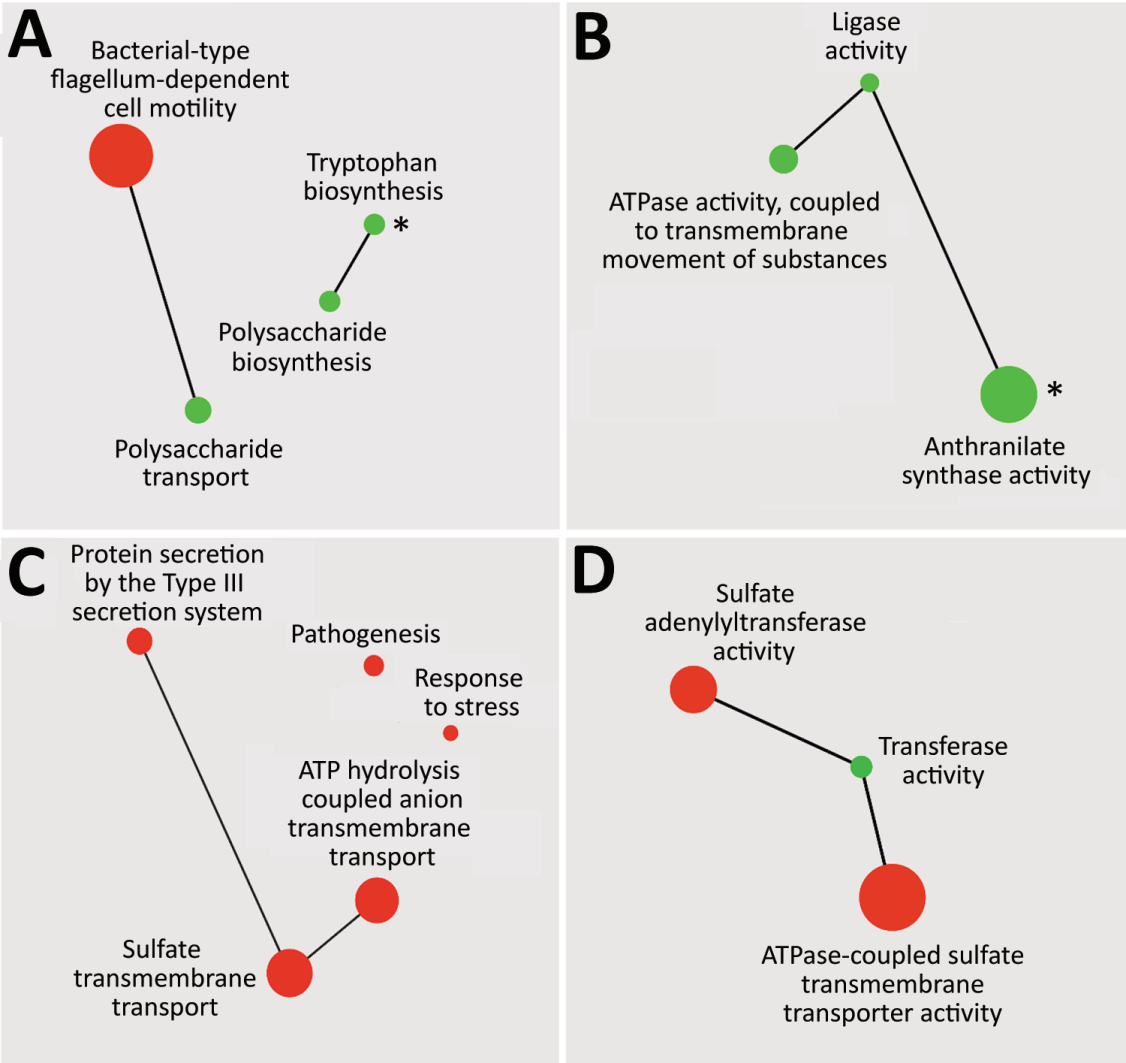
Gene ontology enrichment analysis of genes down-regulated or up-regulated between recent and historic strains of *Bordetella pertussis* from the Czech Republic (A, B) or between both groups of strains from the Czech Republic and Tohama I (C, D). Enriched terms from the domains’ biological process (A, C) and molecular function (B, D) and their catenations, shown as green circles (down-regulated genes) and red circles (up-regulated genes), were summarized by using Revigo (http://revigo.irb.hr) and visualized by using Cytoscape (https://cytoscape.org) as interactive scatter plots. Circle size indicates level of enrichment. Asterisks (*) in panels A and B indicate gene ontology terms that were enriched also for genes down-regulated in recent isolates compared with Tohama I.

Gene ontology enrichment (Figure 4, panel A) showed that within the set of genes, which were significantly modulated between recent and historic isolates, categories such as bacterial type flagellum-dependent cell motility, polysaccharide biosynthesis, and tryptophan biosynthesis were highly enriched. Conversely, when we compared both groups of isolates from the Czech Republic to Tohama I (Figure 4, panel C), genes associated with sulfate transmembrane transport, pathogenesis, and protein secretion by the type III secretion system terms were enriched among the DE genes.

### Clustering of Proteomic Profiles of Recent Isolates, Historic Strains, and Tohama I Strain

**Figure 5:**
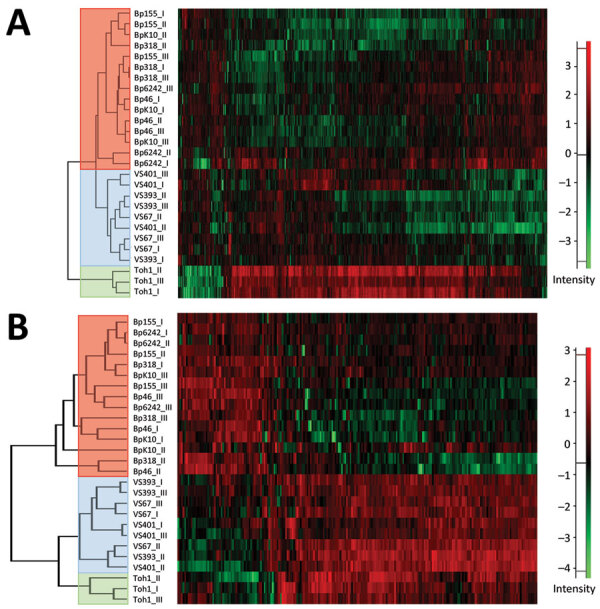
Heatmaps showing hierarchical clustering performed on Z-score normalized log_2_-transformed label-free intensity values of cell-associated (A) or secreted (B) protein fractions of historic and recent isolates of *Bordetella pertussis* from the Czech Republic and the Tohama I strain. Clustering of recent, historic, and Tohama I strains is indicated by red, blue, and green, respectively. Scale bars indicate intensity of proteins normalized by Z-score.

The cell-associated (bacterial pellets) and cell-free (culture supernatants) fractions of selected *B. pertussis* strain cultures were analyzed by using liquid chromatography with mass tandem mass spectrometry. First, hierarchical clustering of the cell-associated protein profiles showed that consistent with RNA sequencing data, strains from the Czech Republic cluster separately from Tohama I and despite high variability among biologic replicates, separation of historic and recent strains was still apparent (Figure 5, panel A). Similarly, hierarchical clustering of secreted proteins indicated that recent isolates cluster apart from historic strains and Tohama I (Figure 5, panel B).

Label-free quantification of cell-associated protein intensities identified 33, 132, and 87 proteins showing significantly changed abundance between recent and historic strains, recent strains and Tohama I, and historic strains and Tohama I, respectively (Appendix 2 Table 5). In good correlation with transcriptomic data, we found that protein levels of hydroxymethylglutaryl-CoA lyase (BP3695), ArsR family transcriptional factor BP2946, small lipoprotein BP2782, and nicotinate-nucleotide diphosphorylase NadC were increased, but levels of several central metabolism enzymes (BP0624–BP0629), tryptophan synthesis genes, and polysaccharide capsule proteins were decreased in recent isolates compared with historic strains (Table 2). Also in support of RNA sequencing data, we determined that various components of the T3SS apparatus and several proteins involved in metabolism of sulfate showed increased abundance compared with Tohama I (Table 3). All strains from the Czech Republic produced pertactin, but flagellar proteins were not detected in any of the studied strains.

**Table 2:** Genes expressing consistently changed RNA and protein levels in recent and historic isolates of *Bordetella pertussis*, Czech Republic*

Gene in Tohama I	Gene group	Gene name	Transcriptome	Proteome	Annotation
toh_00606	Group_2206	BP0624	−2.2	−2.5	Substrate-CoA ligase
toh_00607	Group_2604	BP0625	−2.3	−3.3	Acyl-CoA dehydrogenase
toh_00609	Group_2725	BP0627	−2.0	−3.3	Enoyl-CoA hydratase/isomerase
toh_00610	Group_895	BP0628	−2.4	−2.8	Pyruvate dehydrogenase component
toh_00611	*pdhA*	BP0629	−2.3	−2.8	Pyruvate dehydrogenase component
toh_01576	Group_23	WP_003811211.1	−6.8	−3.3	Capsular biosynthesis protein
toh_01584	*wza*	BP1628	−6.8	−2.2	Capsular polysaccharide export protein
toh_02732	Group_1068	BP2782	−3.9	−3.0	Lipoprotein
toh_02896	Group_128	BP2946	3.6	2.3	*ArsR* family transcriptional regulator
toh_03214	*trpD*	BP3262	−2.1	**−1.4**	Anthranilate phosphoribosyltransferase
toh_03215	*trpG*	BP3263	−2.4	**−1.5**	Anthranilate synthase component II
toh_03216	*trpE*	BP3264	−2.4	**−1.4**	Anthranilate synthase component I
toh_03637	Group_1064	BP3695	3.3	5.5	Hydroxymethylglutaryl-CoA lyase
toh_03667	*nadC*	BP3725	2.5	11.8	Nicotinate-nucleotide diphosphorylase

*Fold change values for recent isolates/historic strains comparison are shown for RNA sequencing and proteomic analyses. Values that did not indicate statistical significance (FC>2; adjusted p<0.05) are shown in bold. toh, Tohama

**Table 3:** Genes expressing consistently increased RNA and protein levels in isolates of *Bordetella pertussis* and 9 infected patients, Czech Republic, compared with Tohama I*

Gene in Tohama I	Gene group	Gene name	Transcriptome			Proteome			Secretome		Annotation
			Cz/Toh	VS/Toh		Cz/Toh	VS/Toh		Cz/Toh	VS/Toh	
toh_00485	Group_1902	BP0500	2.1	2.8		**1.5**	**1.3**		6.0	5.1	T3SS effector BopC
toh_00936	*Sbp*	BP0966	14.7	13.0		5.6	4.6		ND	ND	Sulfate-binding protein
toh_02200	*bscI*	BP2249	2.1	3.1		**1.2**	ND		2.8	6.2	T3SS protein BscI
toh_02203	*bopB*	BP2252	2.1	3.0		3.1	2.8		2.1	1.8	T3SS protein BopB
toh_02204	*bopD*	BP2253	**1.8**	2.5		2.0	1.5		3.7	2.3	T3SS protein BopD
toh_02205	*bcrH1*	BP2254	2.3	4.0		2.5	2.1		**1.6**	**3.1**	T3SS protein
toh_02206	Group_1710	BP2255	2.4	3.8		3.9	4.0		4.8	**1.7**	Hypothetical protein
toh_02207	*bsp22*	BP2256	2.4	3.6		**1.6**	**1.5**		3.6	**1.8**	T3SS protein Bsp22
toh_02208	*bopN*	BP2257	2.5	2.9		**1.3**	**1.0**		3.1	**1.5**	T3SS protein BopN
toh_02210	Group_2630	BP2259	2.0	2.4		**1.4**	**1.9**		5.1	**2.4**	Putative T3SS protein
toh_02214	*bscE*	BP2263	2.3	**1.5**		5.2	4.5		2.0	**2.3**	T3SS protein BscE
toh_03375	Group_1130	BP3434	6.1	4.4		3.3	1.9		2.6	**1.8**	Exported protein

*Fold change values resulting from comparison of either recent isolates (Cz) or historic strains (VS) with Tohama I strain (Toh) are shown for RNA sequencing and proteomic analyses. Values that did not indicate statistical significance (FC>2; adjusted p<0.05) are shown in bold. ND, not determined; toh, Tohama.

Label-free quantification analysis of secreted proteins showed that 121, 130, and 43 proteins displayed significant changes in abundance between recent and historic strains, recent strains and Tohama I, and historic strains and Tohama I, respectively (Appendix 2 Table 6). Levels of several secreted proteins were in good agreement with transcriptomic data (e.g., strains from the Czech Republic and in particular recent isolates secreted increased amounts of several T3SS proteins compared with Tohama I) (Table 3). Abundance of all pertussis toxin subunits and associated transport protein PtlE was higher in recent isolates than in historic strains, which suggests that some of the differences between the *ptxP1* and *ptxp3* strains are also manifested at the level of protein secretion.

### Changes in Genome Structure and Alterations in Gene Expression

Considering the observed differences between historic and recent strains, we attempted to track back the modulated gene expression profiles to alterations in the genome sequence and structure. Because our SNP analysis (Table 2) suggested that there were no SNPs that could explain the altered expression of the DE genes, we have additionally inspected the upstream regions of all DE genes for larger sequence variations. We identified such variations in 4 genes. The gene toh_02779 (*BP2827*) was preceded by an *IS481* element in Bp155, Bp6242, and BpK10, but not in other strains. In addition, 3 DE genes with an upstream *IS481* element had varying gene context further upstream of the transposase in the studied strains (Table 4). Apparently, the observed differences in expression of these genes could be potentially linked to the upstream IS elements.

**Table 4:** Proteins encoded in regions upstream of an *IS481* element adjacent to differentially expressed genes in recent and historic strains of *Bordetella pertussis,* Czech Republic*

Strain	Protein		
	toh_01451 (BP1492)	toh_01915 (BP1969)	toh_02005 (BP2055)
Tohama I	tRNA	Partial phosphonate monoester hydrolase	Partial cyclopropane-fatty-acyl-phospholipid synthase
VS393	tRNA	Partial phosphonate monoester hydrolase	Partial cyclopropane-fatty-acyl-phospholipid synthase
VS401	tRNA	Partial phosphonate monoester hydrolase	IS481 element
VS67	Partial FUSC family protein	Partial phosphonate monoester hydrolase	Partial cyclopropane-fatty-acyl-phospholipid synthase
Bp318	Partial FUSC family protein	MarR family transcriptional regulator	Partial cyclopropane-fatty-acyl-phospholipid synthase
Bp155	Partial FUSC family protein	MarR family transcriptional regulator	Partial cyclopropane-fatty-acyl-phospholipid synthase
Bp46	Partial FUSC family protein	MarR family transcriptional regulator	Partial cyclopropane-fatty-acyl-phospholipid synthase
Bp6242	Partial FUSC family protein	MarR family transcriptional regulator	Partial cyclopropane-fatty-acyl-phospholipid synthase
BpK10	Partial FUSC family protein	MarR family transcriptional regulator	Partial cyclopropane-fatty-acyl-phospholipid synthase

*IS481, insertion sequence 481.

We then tested the possible effect of genome rearrangements on the distance of the DE genes from the origin of replication (*oriC*), a parameter that can greatly affect gene expression (*31*). We determined the distance from *oriC* to all the genes significantly deregulated between recent and historic strains, and although expression of some of the genes inversely correlated with the distance from *oriC*, the differences were not significant.

## Discussion

We conducted a comparative study analyzing the link between genomic organization, gene expression profiles, and protein production/secretion in historic and recent strains of *B. pertussis*. Our results indicate that global changes in genomic structures observed between historic and recent isolates of *B. pertussis* from the Czech Republic translated into different gene expression and protein production profiles. Similarly to other countries, the IS element–driven recombination led to large changes in genomic structures and to considerable gene loss in the isolates from the Czech Republic over the past 50–60 years. Results of our integrated omics analysis support our assumption that genomic rearrangements might affect global expression profiles and phenotypic diversity in *B. pertussis*. Hierarchical clustering of our omics data indicates that strains, which cluster apart at genomic structure level, also have distinct transcriptomic and proteomic profiles.

Given the extent of genome structural variability among both groups of strains, the number of differentially expressed genes was rather low (≈2% of all coding genes). Earlier DNA microarray studies suggested that gene expression profiles between *ptxP1* strains and recent resurgence-associated *ptxP3* lineage differ only subtly (*32**,**33*). Although we have identified an increased number of significantly modulated genes, our data on historic (*ptxP1*) and recent (*ptxP3*) isolates are consistent with these reports. None of the gene expression alterations could be shown to result from nucleotide polymorphism, and only a few could be linked to IS element–induced changes in the local gene context. Upstream of the *IS481* element adjacent to the *BP1492* gene, we identified the *BPt20* tRNA gene in most historic strains and Tohama I, which is, however, missing in recent isolates and historic strain VS67. Thus, it is possible that the activity of the strong tRNA promoter is responsible for increased expression of the *BP1492* gene in historic strains. Also, the presence of an *IS481* element in front of the *BP2827* gene in Bp155, Bp6242, and BpK10 strains might explain the increased expression of this gene in recent isolates. In support of this possibility, when compared with all other strains lacking this IS element, these 3 recent isolates showed highly increased expression of this gene. It is also possible that the observed differences in gene expression between historic and recent strains result from changes in genome organization or gene loss. Bacterial chromosome organization appears to favor a conserved gene order (*34*), and changes in genome architecture and topology can affect gene expression (*35**,**36*). Therefore, it is conceivable that genome rearrangements, resulting in changes in gene order and orientation or in large deletions, might affect transcriptomic profiles in *B. pertussis*.

We have identified 2 previously characterized regions of difference between historic and recent strains, which might offer alternative explanation for the observed differences in gene expression (*20**,**37*). Consistent with these reports, our reports found that genes within RD3 and RD10 are missing in all recent isolates. RD3 contains 2 putative transcriptional regulators (*BP0924* and *BP0928*) of unknown function. Thus, it is probable that absence of these regulators in recent isolates might be accountable for some of the identified alterations in gene expression.

Furthermore, RNA sequencing analysis identified 2 transcriptional regulator genes that are expressed at higher levels in recent isolates, and suggested that some of the observed differences between historic and recent strains might also result from altered expression of regulatory genes. *BP1969*, which encodes a LuxR family transcriptional factor, lies upstream of the *BP1970* and *BP1971* genes, which encode phosphoglucomutase Pgm and phosphoglucose isomerase Pgi. Similarly to *BP1969*, *pgm* and *pgi* genes were significantly up-regulated in recent strains. Therefore, we assume that the *BP1969* gene probably represents a cognate regulator for these glycolytic genes. Besides its role in glycolysis, Pgm catalyzes the generation of sugar nucleotides needed for biosynthesis of lipopolysaccharide and cell wall and was shown to be required for virulence of *B. bronchiseptica* (*38*) and several other pathogens (*39**,**40*). Strains lacking the *pgm* gene showed increased susceptibility to antimicrobial peptides and were attenuated in in vivo models of infection (*38**–**40*). Pgi catalyzes the second step in glycolysis and was shown to be required for virulence of *Xanthomonas* *campestris* (*41*). Thus, we presume that increased expression and production of both enzymes might contribute to increased virulence and fitness of the *ptxP3* lineage.

Among other modulated genes, expression of numerous genes within the operon encoding the flagellar apparatus was significantly increased in recent isolates. However, we could not corroborate this finding because we did not detect any flagellar proteins in our samples. Recent observations suggest that *B. pertussis* is motile under modulatory Bvg-conditions (*42*) and that motility genes are up-regulated during adaptation to the mouse respiratory tract (*43*). Apparently, in vivo conditions, prevailing during *B. pertussis* infections in mice, cannot be completely reproduced under standard laboratory growth conditions, as documented (*43**,**44*), and further experiments are required to determine whether the increased expression translates into higher motility of recent isolates and contributes to improved ability of *ptxP3* strains to colonize the respiratory tract (*33*).

Conversely, expression of an almost complete operon that encodes genes involved in polysaccharide capsule synthesis was substantially down-regulated in recent isolates. This observation is consistent with that of a previous report (*45*) and demonstrates that capsule proteins are produced by *B. pertussis*. This finding also involves the protein responsible for polysaccharide biosynthesis TviD (*BP1618*), which has been reported to be encoded by a pseudogene (*14*).

Data on the role of the capsule in the virulence and physiologic fitness of *B. pertussis* are contradictory. Hoo et al. (*46*) showed that the capsule proteins are expressed during the infection and are required for an efficient colonization of mouse lungs. In contrast, in vitro assays showed that the capsule did not protect *B. pertussis* cells from phagocytosis and serum killing (*45*) and that the capsule locus was not expressed during infection of mouse respiratory tract (*43*). Therefore, it is difficult to assess whether reduced production of capsule proteins provides recent strains with any selective advantage. Nevertheless, the capsular polysaccharides of several gram-negative bacteria are highly immunogenic and were used to formulate carbohydrate–protein conjugate vaccines (*47*). Therefore, it is possible that in circulating isolates of *B. pertussis*, reduced production of the capsule synthesis apparatus contributes to evasion from the host immune response.

Our omics data manifest that, in spite of being isolated at the similar period of time, historic strains are substantially distinct from the reference strain Tohama I. Previous genomic analyses documented that several different clusters of *B. pertussis* circulated in Europe and the United States already in prevaccine and early vaccine eras and that their genomes were different from Tohama I (*20**,**37*). Our results with strains from the Czech Republic are consistent with these observations and also confirm this distinction at the transcriptomic and proteomic levels. For example, expression and production of various sulfate metabolism factors (*sbp*, *cysT*, *cysA*) were strongly reduced in Tohama I compared with strains from the Czech Republic. Likewise, we demonstrated that recent and historic strains had significantly increased expression, production, and secretion of several T3SS components. This observation is consistent with previous reports (*48**,**49*) and confirms that not only recent isolates but also low-passage historic strains of *B. pertussis* are T3SS proficient (*48**,**49*). We conclude that, in agreement with previous reports (*37**,**50*), the Tohama I strain is not a good representative of the circulating *B. pertussis* population.

Collectively, our data suggest that, besides shaping the evolution of *B. pertussis* on a genomic scale, the genome rearrangement and genome reduction processes also affect global transcriptomic and proteomic profiles. In agreement with results of a previous report (*20*), we assume that these mechanisms counterbalance the low level of genetic variability observed in this pathogen and strongly contribute to adaptation of the global population of *B. pertussis*.

Appendix 1 Additional methods on comparative omics analysis of historic and recent isolates of *Bordetella pertussis* and effects of genome rearrangements on evolution.

Appendix 2 Additional data on comparative omics analysis of historic and recent isolates of *Bordetella pertussis* and effects of genome rearrangements on evolution.
