# Supplementary material for: Comparative Omics Analysis of Historic and Recent Isolates of Bordetella pertussis and Effects of Genome Rearrangements on Evolution
Source: Emerg Infect Dis. 2021 Jan;27(1):57–68. doi: 10.3201/eid2701.191541 (PMC7774529; doi:10.3201/eid2701.191541)
Supplement: Appendix 1 — Additional methods on comparative omics analysis of historic and recent isolates of Bordetella pertussis and effects of genome rearrangements on evolution. [file 19-1541-Techapp-s1.pdf]

Article DOI: https://doi.org/10.3201/eid2710.191541

Comparative Omics Analysis of Historic and Recent Isolates of *Bordetella pertussis* and Effects of Genome Rearrangements on Evolution

Appendix 1

Materials and Methods

Whole-Genome Sequencing, Assembly, and Annotation

Genomic DNA was isolated from bacterial cells by using QIAamp UCP Pathogen Mini Kit (QIAGEN, https://www.qiagen.com). Genomes were sequenced on the RSII Platform (PacBio, https://www.pacb.com) and assembled by using the HGAP SMRT Portal protocol (PacBio). Sanger sequencing was used to polish the assemblies. Completed genome sequences were annotated by using Prokka software (*1*) and deposited in the European Nucleotide Archive under project PRJEB26966. For comparison of sequenced strains with the reference strain the genome sequence of Tohama I (strain E476; Appendix Table 1) was reannotated by using our annotation pipeline.

RNA Isolation, Sequencing, and Data Analysis

Total RNA was isolated by using TRI Reagent (Sigma, https://www.sigmaaldrich.com) according to manufacturer’s protocol. Removal of DNA was achieved by treatment of samples with the TURBO DNA-Free Kit (Thermo Fisher Scientific, https://www.thermofisher.com). RNA quality and quantity was determined by agarose gel electrophoresis and using the Nanodrop 2000 Machine (Thermo Fisher Scientific). RNA quality was assessed at sequencing facility by using the 2100 Bioanalyzer Device (Agilent, https://www.agilent.com). All samples displayed RNA integrity numbers >9. Ribosomal RNA was removed by using the Ribo-Zero rRNA Removal Kit for Bacteria (Illumina, https://www.agilent.com). Libraries were prepared by using the Next Ultra II DNA Library Prep Kit (New England Biolabs, https://www.neb.com), and sequenced on an Illumina HiSeq 2500 platform using HiSeqV4 chemistry with single-end 50-bp

reads at the Vienna Biocenter Core Facilities Next Generation Sequencing Unit (Vienna, Austria). After quality control, the reads were demultiplexed and quality trimming and adaptor removal from the reads was performed by using Trimmomatic (*2*).

Reads were mapped to combined transcriptome made of all strains by using the Salmon algorithm (*3*). Combined transcriptome was built from all annotated transcripts in all strains, and homologous transcripts were conflated into multistrain-representing genes. Gene homology between the strains was determined by using the Roary pipeline (*4*). Differential gene expression analysis was performed by using DESeq2 (*5*). Genes with a $\log_2$-fold change $\leq$–1 or $\geq$1 and a q value <0.05 (p value adjusted for multiple testing correction by the method of Benjamini and Hochberg [*6*]) were considered significantly deregulated.

To see the overall gene expression differences between the groups of recent clinical isolates and vaccine strains, all isolates within each group were treated as replicates of the same sample. RNA sequencing data from the sequencing runs were deposited in the European Nucleotide Archive under project accession no. PRJEB34096.

Protein Sample Preparation and Label-Free Proteomic Analysis by Using Liquid Chromatography with Mass Tandem Spectrometry Analyses

Cultures of *B. pertussis* were pelleted by centrifugation at 10,000× *g* at 4°C for 10 min to separate cell pellets and culture supernatants. Cells were resuspended in digestion buffer (100 mmol/L triethylammonium bicarbonate, pH 8.5, 2% sodium deoxycholate) and lysed by sonication. For analysis of supernatant fractions, supernatants were filtered through 0.22-µm filters and precipitated with 10% (wt/vol) trichloracetic acid (Sigma) overnight at 4°C. Precipitated proteins were collected by centrifugation at 14,000× *g* at 4°C, for 20 min, washed with 80% (wt/vol) acetone, and dissolved in 100 mmol/L triethylammonium bicarbonate, pH 8.5, 2% sodium deoxycholate digestion buffer. Protein concentrations were determined by using the BCA Protein Assay Kit (Thermo Fischer Scientific), and 20 µg of protein/sample were used for protein analysis. Cysteines were reduced with 5 mol/L Tris(2-carboxyethyl)phosphine (at 60°C for 60 min) and blocked with 10 mmol/L methyl methanethiosulfonate (at room temperature for 10 min). Samples were digested with trypsin (trypsin:protein ratio 1:20) at 37°C overnight. Digestion of samples was stopped by addition of trifluoracetic acid (Sigma) to a final concentration of 1% (vol/vol). Sodium deoxycholate was removed by extraction with ethyl

acetate, and peptides were desalted on a C18 column (Michrom Bio, https://www.bioprocessonline.com).

A Nano Reversed Phase Column (EASY-Spray Column, 50 cm × 75 μm internal diameter, PepMap C18, 2-μm particles, 100 Å pore size; Thermo Fisher Scientific) was used for liquid chromatography–mass spectrometry analysis. Mobile phase buffer A was composed of water and 0.1% formic acid. Mobile phase B was composed of acetonitrile and 0.1% formic acid. Samples were loaded onto the trap column (Acclaim PepMap300, C18, 5 μm, 300 Å wide pore, 300 μm × 5 mm; Thermo Fisher Scientific) at a flow rate of 15 μL/min. Loading buffer was composed of water, 2% acetonitrile, and 0.1% trifluoroacetic acid. Peptides were eluted with a gradient of phase B ranging from 4% to 35% over 60 min at a flow rate of 300 nL/min. Eluting peptide cations were converted to gas-phase ions by electrospray ionization and analyzed by Orbitrap Fusion (Thermo Fisher Scientific). Survey scans of peptide precursors from 350 m/z to 1,400 m/z were performed at 120 K resolution (at 200 m/z) with a 5×10^5 ion count target.

Tandem mass spectrometry (MS2) was performed by isolation within a 1.5-Th window with the quadrupole, higher-energy collisional dissociation fragmentation with normalized collision energy of 30, and rapid scan mass spectrometry analysis in the ion trap. The MS2 ion count target value was set to 104 and the maximal injection time was 35 ms. The precursors with charge state of 2–6 were sampled for MS2. The dynamic exclusion duration was set to 45 s with a 10 ppm tolerance around the selected precursor and its isotopes. Monoisotopic precursor selection was turned on. The instrument was run at top speed mode with cycles of 2 s (*7*).

Raw data were imported into MaxQuant software version 1.5.3.8 (*8*) for identification and label-free quantification of proteins. The false discovery rate was set to 1% for peptides and minimum specific length of 7 amino acids. The Andromeda search engine (*9*) was used for the MS/MS spectra search against the Uniprot *Bordetella pertussis* Database (https://www.uniprot.org) containing 3,258 entries.

Enzyme specificity was set as C-terminal to Arg and Lys, also enabling cleavage at proline bonds and a maximum of 2 missed cleavages. Dithiomethylation of cysteine was selected as a fixed modification, and N-terminal protein acetylation and methionine oxidation as variable modifications. The match between runs feature of MaxQuant was used to transfer identifications to other liquid chromatography; mass tandem spectrometry runs were based on their masses and

retention time (maximum deviation 0.7 min), and this feature was also used in quantification experiments. Protein abundance was calculated from obtained label-free protein intensities by using he MaxLFQ algorithm (*10*). Proteins with <4 mass tandem spectrometry spectral counts were removed from the analysis. Statistics and data interpretation were performed by using Perseus version 1.6.2.3 software (*11*). Normalized label-free intensities were compared pairwise between recent clinical isolates, vaccine strains, and Tohama I. Similarly to transcriptomic analysis, all isolates within each group of isolates from the Czech Republic were treated as replicates of the same sample, and each abundance ratio was tested for significance by using a 2-group *t*-test ($p<0.05$). The p values were further adjusted for multiple testing correction to control the false-discovery rate at a cutoff value of 0.05 by using the permutation test (number of randomizations = 250). Proteins with corrected p values (q values) <0.05 were considered as significantly modulated. For downstream analyses (e.g., gene ontology [GO] term enrichment), only proteins that were detected by ≥ 2 unique peptides in ≥ 2 of the 3 biologic replicates were considered. Proteins for which label-free intensities were not obtained in any of the replicates of either the vaccine strains, clinical isolates, or the Tohama I strain were considered as strongly modulated and defined as ON/OFF. Proteomics data were deposited in the ProteomeXchange Consortium by using the PRIDE (*12*) partner repository with the dataset identifier PXD015184.

The hierarchical clustering analysis was generated by using Perseus 1.6.2.3 software (*11*). In brief, intensities of label-free quantified proteins were log2-transformed to reduce the effect of outliers. For analysis, the greatly modulated proteins were separated by using a multiple-sample test with the false discovery rate at a cutoff value of 0.05 by using the permutation test (250 randomizations). Hierarchical clustering was performed on Z-score normalized log2-transformed label-free quantified intensities of greatly modulated proteins within either cell-associated or cell-free fractions.

GO Term Enrichment Analysis

To gain a comprehensive functional annotation of the reference genome, GO terms per gene were deduced by using blast2go (*13*). For the GO term enrichment analysis, deregulated genes were split into up-regulated and down-regulated genes, and each gene set was analyzed separately. Each GO term, which was associated with >1 gene in the gene set, was tested for enrichment in comparison to the whole transcriptome by using the Fisher exact test. Afterwards, determined p values were corrected for multiple testing by using the method of Benjamini and

Hochberg (*6*), summarized by using the Revigo tool (*14*), and visualized by using Cytoscape (https://cytoscape.org).

References

1. Seemann T. Prokka: rapid prokaryotic genome annotation. Bioinformatics. 2014;30:2068–9. PubMed https://doi.org/10.1093/bioinformatics/btu153

2. Bolger AM, Lohse M, Usadel B. Trimmomatic: a flexible trimmer for Illumina sequence data. Bioinformatics. 2014;30:2114–20. PubMed https://doi.org/10.1093/bioinformatics/btu170

3. Patro R, Duggal G, Love MI, Irizarry RA, Kingsford C. Salmon provides fast and bias-aware quantification of transcript expression. Nat Methods. 2017;14:417–9. PubMed https://doi.org/10.1038/nmeth.4197

4. Page AJ, Cummins CA, Hunt M, Wong VK, Reuter S, Holden MT, et al. Roary: rapid large-scale prokaryote pan genome analysis. Bioinformatics. 2015;31:3691–3. PubMed https://doi.org/10.1093/bioinformatics/btv421

5. Love MI, Huber W, Anders S. Moderated estimation of fold change and dispersion for RNA-seq data with DESeq2. Genome Biol. 2014;15:550. PubMed https://doi.org/10.1186/s13059-014-0550-8

6. Benjamini Y, Hochberg Y. Controlling the false discovery rate: a practical and powerful approach to multiple testing. J R Stat Soc B. 1995;57:289–300. https://doi.org/10.1111/j.2517-6161.1995.tb02031.x

7. Hebert AS, Richards AL, Bailey DJ, Ulbrich A, Coughlin EE, Westphall MS, et al. The one hour yeast proteome. Mol Cell Proteomics. 2014;13:339–47. PubMed https://doi.org/10.1074/mcp.M113.034769

8. Cox J, Mann M. MaxQuant enables high peptide identification rates, individualized p.p.b.-range mass accuracies and proteome-wide protein quantification. Nat Biotechnol. 2008;26:1367–72. PubMed https://doi.org/10.1038/nbt.1511

9. Cox J, Neuhauser N, Michalski A, Scheltema RA, Olsen JV, Mann M. Andromeda: a peptide search engine integrated into the MaxQuant environment. J Proteome Res. 2011;10:1794–805. PubMed https://doi.org/10.1021/pr101065j

10. Cox J, Hein MY, Luber CA, Paron I, Nagaraj N, Mann M. Accurate proteome-wide label-free quantification by delayed normalization and maximal peptide ratio extraction, termed MaxLFQ. Mol Cell Proteomics. 2014;13:2513–26. PubMed https://doi.org/10.1074/mcp.M113.031591

11. Tyanova S, Temu T, Sinitcyn P, Carlson A, Hein MY, Geiger T, et al. The Perseus computational platform for comprehensive analysis of (prote)omics data. Nat Methods. 2016;13:731–40. PubMed https://doi.org/10.1038/nmeth.3901

12. Perez-Riverol Y, Csordas A, Bai J, Bernal-Llinares M, Hewapathirana S, Kundu DJ, et al. The PRIDE database and related tools and resources in 2019: improving support for quantification data. Nucleic Acids Res. 2019;47(D1):D442–50. PubMed https://doi.org/10.1093/nar/gky1106

13. Conesa A, Götz S, García-Gómez JM, Terol J, Talón M, Robles M. Blast2GO: a universal tool for annotation, visualization and analysis in functional genomics research. Bioinformatics. 2005;21:3674–6. PubMed https://doi.org/10.1093/bioinformatics/bti610

14. Supek F, Bošnjak M, Škunca N, Šmuc T. REVIGO summarizes and visualizes long lists of gene ontology terms. PLoS One. 2011;6:e21800. PubMed https://doi.org/10.1371/journal.pone.0021800
